# High Mobility Ge pMOSFETs with ZrO_2_ Dielectric: Impacts of Post Annealing

**DOI:** 10.1186/s11671-019-3037-4

**Published:** 2019-06-11

**Authors:** Huan Liu, Genquan Han, Yan Liu, Yue Hao

**Affiliations:** State Key Discipline Laboratory of Wide Band Gap Semiconductor Technology, School of Microelectronics, Xidian University, Xi’an, 710071 China

**Keywords:** Germanium, MOSFET, ZrO_2_, PMA, PDA, Mobility

## Abstract

This paper investigates the impacts of post metal annealing (PMA) and post deposition annealing (PDA) on the electrical performance of Ge p-type metal-oxide-semiconductor field-effect transistors (pMOSFETs) with ZrO_2_ dielectric. For the transistors without PDA, on-state current (*I*_ON_), subthreshold swing (SS), and capacitance equivalent thickness (CET) characteristics are improved with PMA temperature increasing from 350 to 500 °C. Crystallization of ZrO_2_ dielectric at the higher PMA temperature contributes to the increase of the permittivity of ZrO_2_ and the decrease of the density of interface states (*D*_it_), resulting in a reduced CET and high effective hole mobility (*μ*_eff_). It is demonstrated that Ge pMOSFETs with a PDA treatment at 400 °C have a lower CET and a steeper SS but a lower *μ*_eff_ compared to devices without PDA.

## Background

Germanium (Ge) has been regarded as one of the attractive p-channel materials for advanced CMOS because it offers much higher hole mobility than does Si [1–3]. A high-quality gate dielectric and effective passivation of Ge surface are the keys to realizing the superior effective carrier mobility (*μ*_eff_) and high drive current in Ge transistor [4–7]. Several high-κ materials such as HfO_2_ [8], ZrO_2_ [7, 9], La_2_O_3_ [10], and Y_2_O_3_ [11] have been studied as the alternative gate dielectrics for Ge p-type metal-oxide-semiconductor field-effect transistors (pMOSFETs) to achieve capacitance equivalent thickness (CET) scalability toward sub-1 nm. Among these, ZrO_2_ dielectric has attracted most attention due to the much higher κ value [12, 13] and the better interfacial quality [14] compared to the Hf-based ones. It has widely been reported that crystallization of ZrO_2_ can further improve the electrical performance of Ge pMOSFET, e.g., reducing CET and boosting *μ*_eff_ [15, 16]. However, there is a lack of study on the impacts of process steps for ZrO_2_ crystallization on device performance of Ge transistors.

In this paper, we investigate the impacts of the post metal annealing (PMA) and the post deposition annealing (PDA) on the electrical performance of Ge pMOSFETs with ZrO_2_ dielectric. Significantly improved *μ*_eff_ and reduced CET can be achieved in devices at higher PMA temperature.

## Methods

**Fig. 1:**
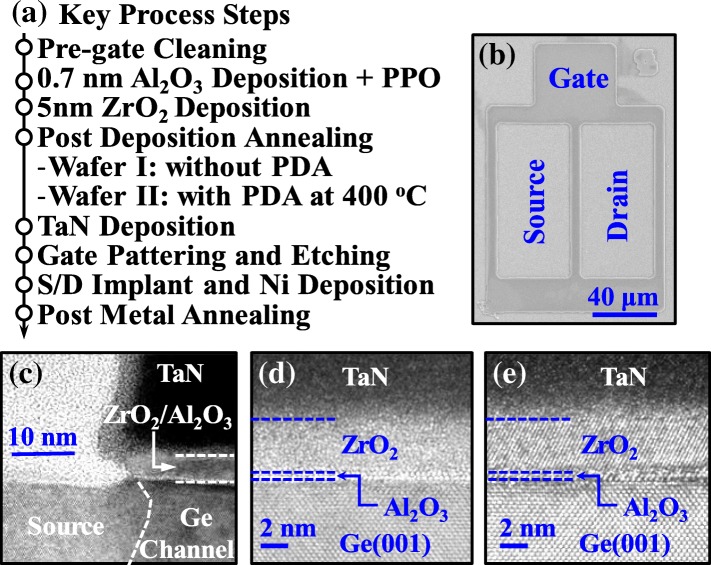
**a** Key process steps for fabricating Ge pMOSFETs with ZrO_2_ dielectric. **b** SEM image of the fabricated transistor. **c** XTEM image of Ge pMOSFET showing the gate and S/D regions. **d**, **e** HRTEM images of gate stacks of Ge pMOSFETs on wafer I annealed at 400 °C and 500 °C, respectively

Key process steps for fabricating Ge pMOSFETs with ZrO_2_ dielectric are shown in Fig. 1a. The Ge pMOSFETs were fabricated on n-type Ge(001) wafer with a resistivity of 0.088–0.14 Ω∙cm. After the several cycles of chemical cleaning in the diluted HF (1:50) solution and rinsing in DI water. Ge wafer was loaded into an atomic layer deposition (ALD) chamber. The Ge surface was passivated by an ozone post oxidation (OPO), i.e., an ultrathin Al_2_O_3_ layer was deposited at 300 °C, and then, the in situ OPO was carried out at 300 °C for 15 min. After that, a 5-nm-thick ZrO_2_ was deposited at 250 °C in the same ALD chamber using TDMAZr and H_2_O as precursors of Zr and O, respectively. During the deposition, Zr[N(CH_3_)_2_]_4_ source was heated to 85 °C. PDA process was carried out on some sample at 400 °C for 60 s using the rapid thermal annealing. Samples with and without PDA were denoted wafer II and I, respectively. Then, a 100-nm-thick TaN gate electrode was deposited by reactive sputtering. After the gate patterning and etching, the source/drain (S/D) regions were formed by BF_2_^+^ implantation at an energy of 30 keV and a dose of 1 × 10^15^ cm^−2^. Fifteen-nanometer nickel S/D contacts were formed by a lift-off process. Finally, the PMA at 350, 400, 450, and 500 °C for 30 s was carried out for dopant activation and S/D metallization.

Figure 1b shows the scanning electron microscope (SEM) image of a fabricated Ge pMOSFET. Figure 1c shows the cross-sectional transmission electron microscope (XTEM) image of Ge pMOSFET, showing the source/drain region, metal gate, and ZrO_2_ dielectric. Figure 1d and e show the high-resolution TEM (HRTEM) images of the gate stacks of Ge pMOSFETs with a PMA at 400 and 500 °C, respectively, on wafer I. It is observed that the ZrO_2_ dielectric was fully crystallized and underwent a PMA at 500 °C. The thickness of Al_2_O_3_ interfacial layer is about 0.7 nm.

## Results and Discussion

**Fig. 2:**
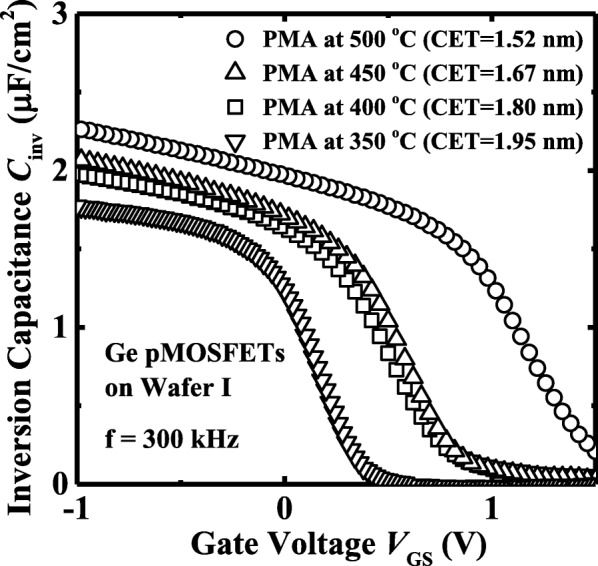
Inversion *C*_inv_-*V*_GS_ curves for the Ge pMOSFETs on wafer I with a PMA at 350 °C, 400 °C, 450 °C, and 500 °C

Inversion capacitance *C*_inv_ vs. *V*_GS_ curves measured at a frequency of 300 kHz for the devices on wafer I are shown in Fig. 2. The CET values are extracted to be ∼ 1.95, 1.80, 1.67, and 1.52 nm for the devices with PMA at 350, 400, 450, and 500 °C, respectively. The smaller CET is achieved at a higher PMA temperature due to the crystallization of ZrO_2_. In general, the κ values for amorphous and crystalline ZrO_2_ are about 20–23 and 28–30, respectively. A 5-nm-thick crystalline ZrO_2_ contributes an EOT of ~ 0.7 nm. The shift of *C-V* curves with various PMA temperature is due to the fact that crystallization reduces the density of bulk traps in ZrO_2_ dielectric.

**Fig. 3:**
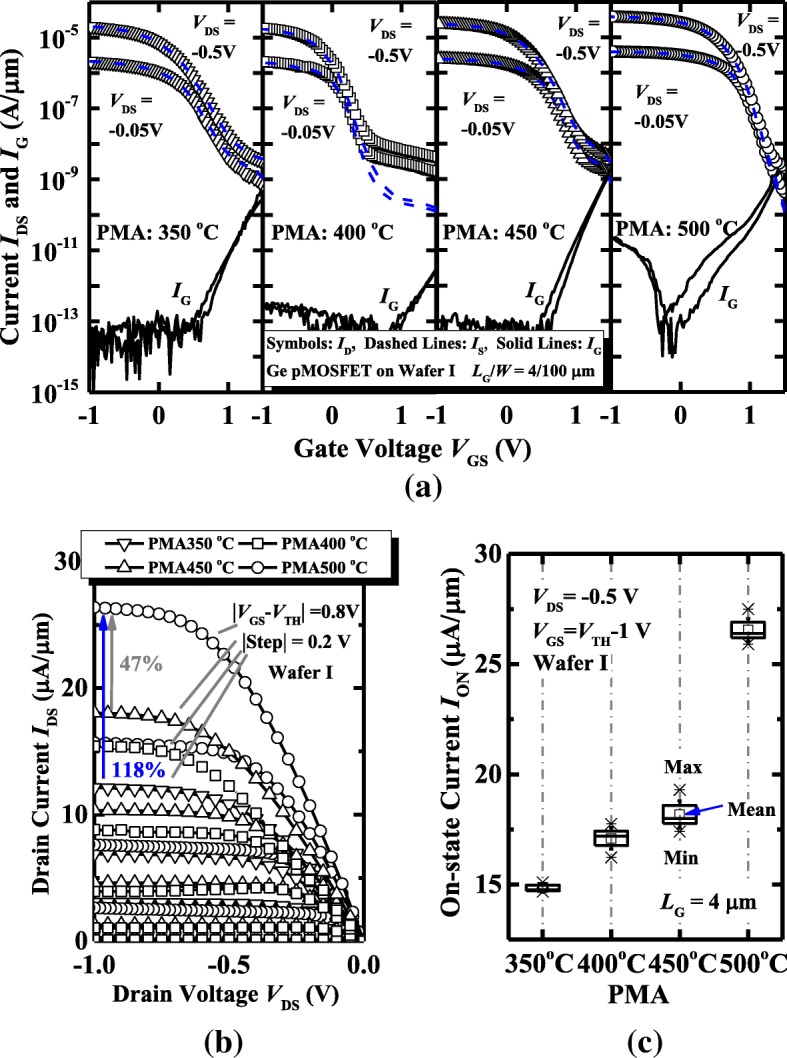
**a** Measured *I*_D_, *I*_S_, and *I*_G_ *vs. V*_GS_ curves of Ge pMOSFETs on wafer I with the PMA at 350, 400, 450, and 500 °C. **b** *I*_DS_-*V*_DS_ curves measured at the different *V*_GS_-*V*_TH_ for the devices. **c** Device annealed at 500 °C has a higher on-state current *I*_ON_ compared to the transistors with the PMA at the lower temperatures

Figure 3a shows the measured transfer characteristics and gate leakage currents *I*_G_ of Ge pMOSFETs on wafer I with the different PMA temperatures. All the devices have a gate length *L*_G_ of 4 μm and a gate width *W* of 100 μm. Ge pMOSFETs exhibit the much lower *I*_G_ compared to *I*_DS_ for all the PMA temperatures. An *I*_ON_/*I*_OFF_ ratio above 10^4^ is achieved for the device with a PMA at 500 °C. The *I*_DS_-*V*_DS_ curves of the devices measured at the different gate overdrive |*V*_GS_-*V*_TH_| are shown in Fig. 3b. It is noted that the threshold voltage *V*_TH_ is defined as the *V*_GS_ at *I*_DS_ of 10^−7^ A/μm. The Ge transistor with a PMA at 500 °C obtains the ~ 47% and 118% drive current improvement compared to the devices annealed at 450 °C and 350 °C, respectively, at a *V*_DS_ of − 1.0 V and a |*V*_GS_-*V*_TH_| of 0.8 V. Figure 3c shows the statistical plot of the *I*_ON_ at a *V*_DS_ of − 0.5 V and a *V*_GS_-*V*_TH_ of − 1 V for Ge pMOSFETs with the various PMA temperatures. All the transistors in this plot have an *L*_G_ of 4 μm and a *W* of 100 μm. Devices with a PMA at 500 °C exhibit an improved *I*_ON_ as compared to those with the lower PMA temperatures, which is attributed to the decreased S/D resistance, the reduced CET, and the higher *μ*_eff_, which will be discussed later.

**Fig. 4:**
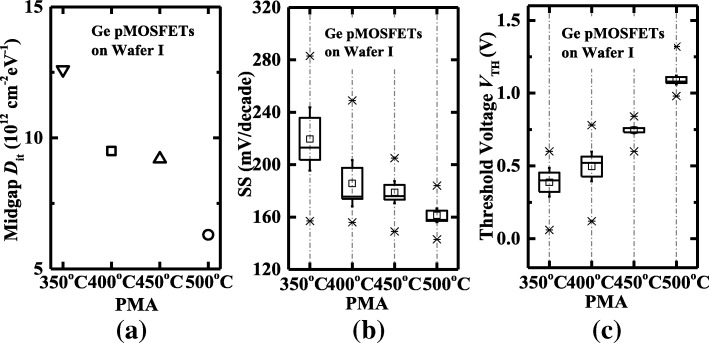
Comparison of **a** midgap *D*_it_, **b** SS, and **c** *V*_TH_ for Ge pMOSFETs on wafer I with the PMA at 350, 400, 450, and 500 °C

Figure 4 shows the statistical plots of midgap *D*_it_, SS, and *V*_TH_ characteristics for the devices with the different PMA temperatures. As shown in Fig. 4a, based on the maximum conductance method [17], the midgap *D*_it_ values are extracted to be 1.3 × 10^13^, 9.5 × 10^12^, 9.2 × 10^12^, and 6.3 × 10^12^ cm^−2^ eV^−1^ for the devices with the PMA at 350, 400, 450, and 500 °C, respectively. Figure 4b presents that Ge pMOSFETs annealed at 500 °C have the improved SS characteristics than the transistors annealed at the lower temperatures, due to the smaller midgap *D*_it_ and CET. The values of *D*_it_ and SS of Ge pMOSFETs with PMA are still higher than those of the best reported Ge transistors. It could possibly be reduced by optimizing the OPO passivation module, e.g., Al_2_O_3_ thickness and ozone oxidation temperature and duration. *V*_TH_ shifts to the positive *V*_GS_ with the increasing of PMA temperature, which is originated from the reduced CET and *D*_it_. It is concluded that the best electrical performance is achieved for Ge pMOSFETs with a PMA at 500 °C.

**Fig. 5:**
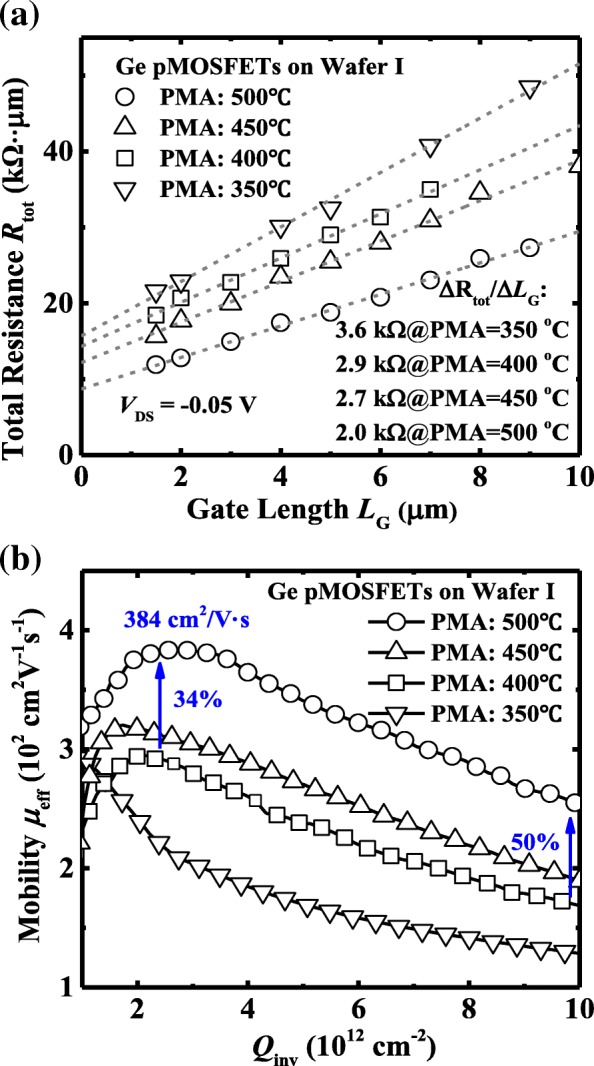
**a** *R*_tot_ as a function of *L*_G_ at a *V*_GS_-*V*_TH_ of − 1 V and a *V*_DS_ of − 0.05 V for devices on wafer I with various PMA temperatures. **b** *μ*_eff_ *vs. Q*_inv_ extracted by the split *C*-*V* method. The highest mobility is obtained in devices with a PMA at 500 °C

*μ*_eff_, as a crucial factor affecting device drive current and transconductance in Ge pMOSFETs, was measured using the Δ*R*_tot_/Δ*L*_G_ method [18]. A large number of devices were measured with *L*_G_ ranging from 1.5 to 9 μm. Figure 5a illustrates the total resistance *R*_tot_ extracted at a |*V*_GS_-*V*_TH_| of − 1 V and a *V*_DS_ of − 0.05 V as a function of *L*_G_. The *R*_SD_ is the value at which the fitted line intersects at the *y*-axis. The *R*_SD_ values were estimated about to be 7.85, 7.15, 6.10, and 4.35  kΩ ·μm for devices with PMA at 350, 400, 450, and 500 °C, respectively. This is indicative of the better dopant activation of S/D at higher PMA temperature. *μ*_eff_ can be extracted by *μ*_eff_ = 1/[*WQ*_inv_(Δ*R*_tot_/Δ*L*_G_)], where *Q*_inv_ is the inversion charge density in Ge channel and Δ*R*_tot_/Δ*L*_G_ is the slope of the *R*_tot_ vs. *L*_G_ as shown in Fig. 5a. The smaller Δ*R*_tot_/Δ*L*_G_ for devices with PMA at 500 °C indicates an enhancement in *μ*_eff_ as compared with transistors with PMA at 450 °C. Figure 5b shows *μ*_eff_ as a function of *Q*_inv_ curves, extracted using the split *C*-*V* method. The peak hole mobility is 384 cm^2^/V ·s for devices with a PMA at 500 °C, which is 31% higher than that of the devices with a PMA at 400 °C. At a high *Q*_inv_ of 1 × 10^13^ cm^−2^, Ge pMOSFETs which underwent a PMA at 500 °C achieve a mobility enhancement in comparison with the devices annealed at 400 °C. Ge transistors with crystalline ZrO_2_ have the lower density of bulk trap charge resulting in the lower remote Coulomb scattering of holes, compared to the devices with amorphous ZrO_2_. Owing to the smooth interface between crystalline ZrO_2_ and Ge, Ge devices annealed at 500 °C have a lower surface roughness scattering and show a shift of peak mobility to the higher *Q*_inv_.

**Fig. 6:**
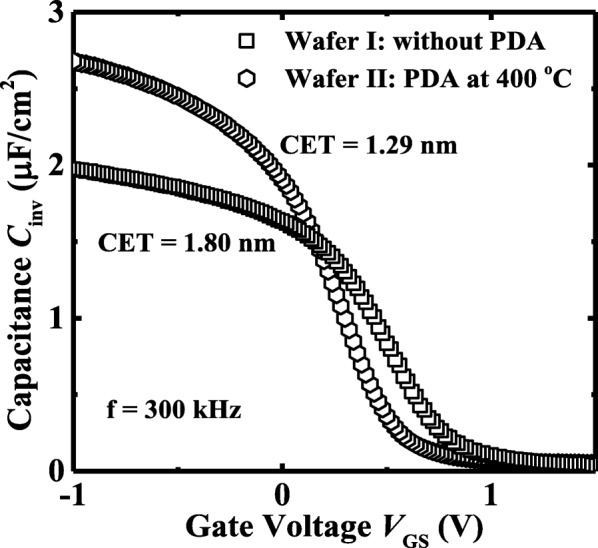
*C*_inv_*-V*_GS_ plots for the devices on wafer I and II with a PMA at 400 °C

**Fig. 7:**
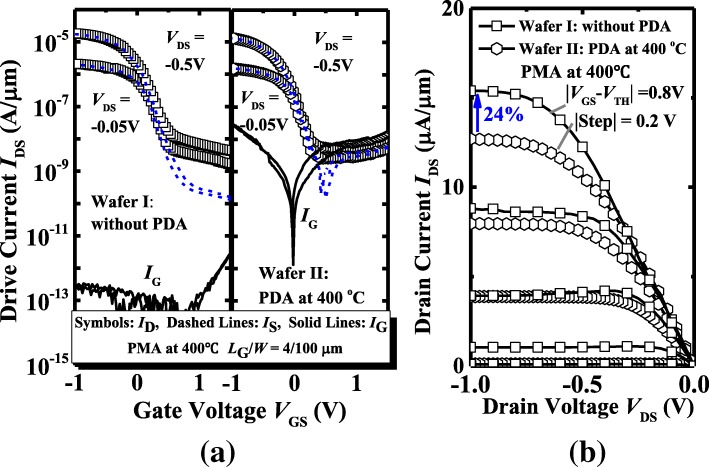
**a** *I*_D_, *I*_S_, and *I*_G_ *vs. V*_GS_ curves of Ge pMOSFETs on wafer I and II with PMA at 400 °C. **b** *I*_DS_-*V*_DS_ curves measured at different *V*_GS_-*V*_TH_ for the devices

Next, we discuss the impacts of PDA on the electrical characteristics of Ge pMOSFETs. Figure 6 shows the measured *C*_inv_ vs. *V*_GS_ of the Ge pMOSFETs on wafer I and wafer II with a PMA at 400 °C. The device which underwent a PDA at 400 °C has a much lower CET value of 1.29 nm compared to the device without PDA, 1.80 nm. Figure 7a shows the *I*_D_, *I*_S_, and *I*_G_-*V*_GS_ characteristic curves of Ge pMOSFETs on wafer I and wafer II, and the devices which underwent a PMA at 400 °C. A larger gate leakage current is obtained for the device with PDA compared to the transistor without PDA, which is due to the lower CET. The corresponding *I*_DS_-*V*_DS_ curves of the devices measured at different gate overdrive *V*_GS_-*V*_TH_ are shown in Fig. 7b. The Ge transistor without PDA shows a ~ 24% improvement in drive current over the one with PDA at 400 °C at the same overdrive of − 0.8 V in the saturation region.

**Fig. 8:**
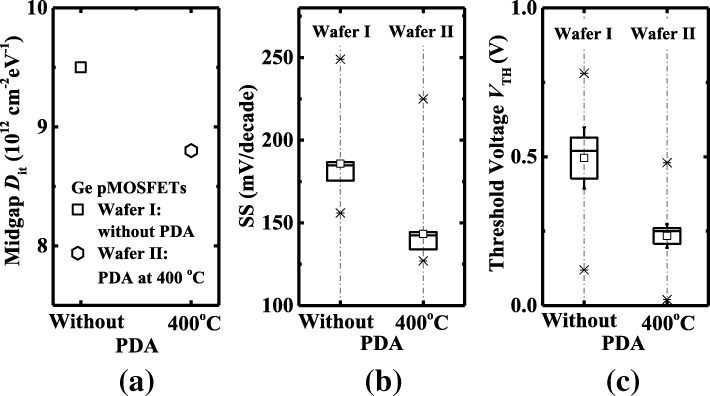
Comparison of **a** midgap *D*_it_, **b** SS, and **c** *V*_TH_ for Ge pMOSFETs on wafer I and II with PMA at 400 °C

Figure 8 plots the statistical results of midgap *D*_it_, SS, and *V*_TH_ of the Ge pMOSFETs with and without PDA. Figure 8a shows that the smaller *D*_it_ is achieved in Ge pMOSFETs with PDA at 400 °C compared to devices without PDA. In Fig. 8b, the lower value of mean subthreshold swing of 142 mV/decade is achieved for devices with PDA at 400 °C, corresponding to the lower CET and the lower *D*_it_. It indicates that devices with PDA at 400 °C have a superior ZrO_2_/Ge interface. Figure 8c shows that devices with and without PDA have a different *V*_TH_; it may be attributed to the density of traps in the lower bandgap half dominant in the *V*_TH_.

**Fig. 9:**
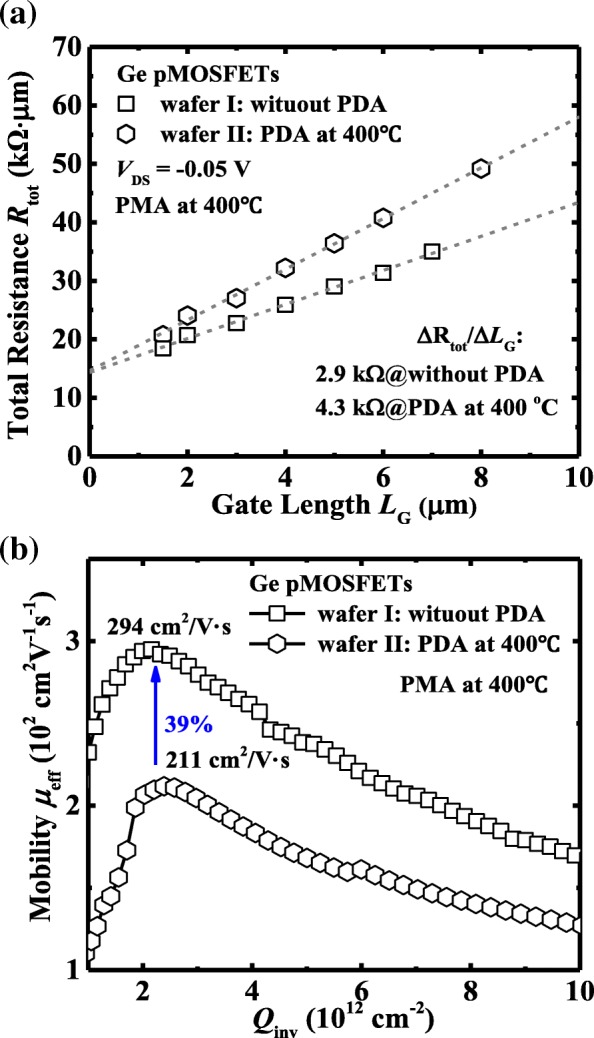
**a** *R*_tot_ vs. *L*_G_ curves for devices on wafer I and wafer II with PMA at 400 °C. **b** Hole mobility *μ*_eff_ vs. *Q*_inv_ for devices with and without PDA

Figure 9a shows the *R*_tot_ vs. *L*_G_ curves at a gate overdrive of − 1 V and *V*_DS_ of − 0.05 V for devices with a PMA at 400 °C. The *R*_SD_ values are estimated about to be 7.15 and 7.30 kΩ·μm for devices without and with PDA at 400 °C, respectively. As shown in Fig. 9b, a remarkable higher peak *μ*_eff_ is achieved for Ge pMOSFETs without PDA, corresponding the smaller Δ*R*_tot_/Δ*L*_G_ in Fig. 9a, compared to devices with PDA. The devices with a PDA at 400 °C exhibit a peak *μ*_eff_ of 211 cm^2^/V·s; the lower hole mobility might be mainly attributed to the strong remote Coulomb scattering contributed by the fixed charge in ZrO_2_ dielectric.

## Conclusions

In summary, the impacts of PMA and PDA on Ge pMOSFET with ZrO_2_ dielectric were extensively investigated. Crystallization of ZrO_2_ gate dielectric provides for significantly enhanced hole mobility and reduced CET compared to devices at the lower PMA temperature. A peak hole mobility of 384 cm^2^/V·s and enhanced drive current have been achieved in devices with PMA at 500 °C. Devices with PDA at 400 °C exhibited the lower CET and the smaller *D*_it_ but the poor hole mobility and the larger leakage current compared with transistors without PDA.

## Data Availability

The datasets supporting the conclusions of this article are included in the article.

## Abbreviations

ALD: Atomic layer deposition
BF_2_^+^: Boron fluoride ion
CET: Capacitive effective thickness
Ge: Germanium
HF: Hydrofluoric acid
HRTEM: High-resolution transmission electron microscope
IL: Interfacial layer
MOSFETs: Metal-oxide-semiconductor field-effect transistors
Ni: Nickel
PDA: Post deposition annealing
PMA: Post metal annealing
SS: Subthreshold swing
TaN: Tantalum nitride
TDMAZr: Tetrakis (dimethylamido) hafnium
ZrO_2_: Zirconium dioxide
*μ*_eff_: Effective carrier mobility
